# Culturable Heavy Metal-Resistant and Plant Growth Promoting Bacteria in V-Ti Magnetite Mine Tailing Soil from Panzhihua, China

**DOI:** 10.1371/journal.pone.0106618

**Published:** 2014-09-04

**Authors:** Xiumei Yu, Yanmei Li, Chu Zhang, Huiying Liu, Jin Liu, Wenwen Zheng, Xia Kang, Xuejun Leng, Ke Zhao, Yunfu Gu, Xiaoping Zhang, Quanju Xiang, Qiang Chen

**Affiliations:** 1 Department of Microbiology, College of Resource and Environmental Sciences, Sichuan Agricultural University, Chengdu, China; 2 Environmental Monitoring Station, Panzhihua Municipal Environmental Protection Bureau, Panzhihua, China; NERC Centre for Ecology & Hydrology, United Kingdom

## Abstract

To provide a basis for using indigenous bacteria for bioremediation of heavy metal contaminated soil, the heavy metal resistance and plant growth-promoting activity of 136 isolates from V-Ti magnetite mine tailing soil were systematically analyzed. Among the 13 identified bacterial genera, the most abundant genus was *Bacillus* (79 isolates) out of which 32 represented *B. subtilis* and 14 *B. pumilus*, followed by *Rhizobium* sp. (29 isolates) and *Ochrobactrum intermedium* (13 isolates). Altogether 93 isolates tolerated the highest concentration (1000 mg kg^−1^) of at least one of the six tested heavy metals. Five strains were tolerant against all the tested heavy metals, 71 strains tolerated 1,000 mg kg^−1^ cadmium whereas only one strain tolerated 1,000 mg kg^−1^ cobalt. Altogether 67% of the bacteria produced indoleacetic acid (IAA), a plant growth-promoting phytohormone. The concentration of IAA produced by 53 isolates was higher than 20 µg ml^−1^. In total 21% of the bacteria produced siderophore (5.50–167.67 µg ml^−1^) with two *Bacillus* sp. producing more than 100 µg ml^−1^. Eighteen isolates produced both IAA and siderophore. The results suggested that the indigenous bacteria in the soil have beneficial characteristics for remediating the contaminated mine tailing soil.

## Introduction

Mining industry has caused extensive environmental and public health problems [1]–[3]. A wide variety of heavy metals such as zinc, lead, copper and cadmium have been detected in soil at mining sites presenting a major threat to the environment and population [4], [5]. Heavy metals cannot be biologically degraded and indefinitely persist in the environment. Heavy metals transferred through the food chain are a serious hazard to human health [6]. Due to contamination by heavy metals, mining sites are surrounded by large barren areas. The awareness of the detrimental heavy metal contamination at mining sites has increased in recent years.

The toxic heavy metals accumulated in soil can effectively impact the microbial community composition. Bacteria play an important role in maintaining soil fertility and structure. Because bacteria respond quickly and are sensitive to subtle environmental changes, they have been considered as efficient bio-indicators of soil quality [7]. Both the structural and functional bacterial diversity are important indicators of soil health [8]. Phytoremediation has been effectively used to remediate heavy metal-polluted sites as a sustainable remediation approach [9]. Plant-microbe partnerships may be utilized to improve biomass production and remediation [10]. Plant growth-promoting rhizobacteria (PGPR) that solubilize phosphate and synthesize growth-promoting substances such as indoleacetic acid (IAA) and siderophores can be applied in the plant-assisted bioremediation of metal-contaminated soil [11]–[14]. Phytoremediation utilizes heavy metal-tolerant plant species with metal accumulation ability. Since the addition of IAA to soil can enhance the uptake of metals in plant roots [15], [16], bacteria-producing IAA have been used to assist the phytoremediation of soil contaminated with heavy metals [17].

Metals such as iron, zinc, copper, manganese and nickel play important roles as essential or beneficial micronutrients of microorganisms [18], [19]. However, a high concentration of metal ions in soil shows serious effects on microbial communities by changing the community structure and decreasing diversity and total microbial biomass [13]. Therefore, microbial communities are useful indicators of the effect of contamination on soil health [20]. To have a functional role in remediation, bacteria in heavy metal-contaminated soil must first overcome the heavy metal stress. Microorganisms tolerate heavy metals by immobilizing metals on cell surfaces or transforming metals into less toxic forms, for example by precipitation, acidification and oxidation-reduction [21].

Panzhihua is an industrial and mining city in Sichuan of Southwest China with over 10^9^ tonnes of ore reserves deposited as iron-vanadium-titanium oxide (V-Ti magnetite) [22]. The world class magmatic deposits of V-Ti magnetite in Panzhihua provide 20% of iron (Fe), 64% of vanadium (V) and 53% of titanium (Ti) supply for China [23]. Long-term mining activities have contaminated soils and sediments in Panzhihua by metals, especially by V, Ti and Fe [24]. The redox-sensitive vanadium is toxic to soil microorganisms and plants [25]. Even though titanium is beneficial to plants at low concentrations, high concentrations of titanium are toxic [26]. Iron is an essential nutrient serving as a catalyst for many cellular reactions, in particular those involving redox and O_2_ chemistry [27]. More than 220 million m^3^ of mine tailing has been piled up in Zhujiabaobao, Panzhihua, creating a serious environmental hazard. Because the mine tailing soil contains heavy metals, only few plants grow on it, creating large barren areas. Long term exposure to contaminant allows different bacteria to become adapted to the contaminant, making autochthonous bacteria more useful in bioremediating the contaminated environment compared to allochthonous ones [28]. Therefore, this study focuses on the culturable heavy metal-resistant and plant growth-promoting autochthonous bacteria from V-Ti magnetite mine tailing soil, with the aim of providing information for bioremediating the large area covered by the V-Ti magnetite mine tailing dam at Panzhihua.

## Materials and Methods

### Study site and soil sampling

Panzhihua (N26°05′∼27°21′, E101°08′∼102°15′) in Sichuan, China, is an important industrial and mining base with abundant mineral resources. Panzhihua V-Ti magnetite mine is by the Jinsha River in the southern part of Panxi rift valley upstream of the Yangtze River. The mining area includes six large scale iron deposits, numerous medium-sized coal, clay, dolomite and limestone deposits, and minor graphite, manganese and barite deposits [23]. Our study site did not involve endangered or protected species and provide a specific location, so no specific permissions were required for the location/activity.

Zhujiabaobao (N 26°37′2″, E 101°45′56″) in Panzhihua is a huge deposit of V-Ti magnetite. In the Zhujiabaobao mining area, there is more than 220 million m^3^ of mine tailing and a large tailing dam. The ground around the tailing dam is barren and desert-like. Four soil samples were collected in May 2013 to a depth of 0–20 cm from the tailing dam by a five-point sampling method. Soil samples, fully mixed in sterile polyethylene bags, were kept at room temperature in the dark.

### Chemical analyses of soil samples

The soil samples were dispersed and passed through a 2 mm sieve before measuring pH, organic matter content and the concentrations of available nitrogen (N), phosphorus (P) and potassium (K) as described [29]. Soil available N was determined by Aalkali N-proliferation method, whereas and available P and K quantified with the ASI method. Soil organic matter was determined by the K_2_CrO_7_−H_2_SO_4_ oxidation method. To extract heavy metals, air-dried soil samples were passed through a 2 mm nylon sieve, and digested by 1∶2∶2 (V:V:V) HNO_3_:HCl:HClO_4_. Vanadium (V), titanium (Ti), iron (Fe), nickel (Ni), lead (Pb), zinc (Zn), manganese (Mn), copper (Cu), arsenic (As), cadmium (Cd) and chromium (Cr) were measured by inductively coupled plasma atomic emission spectroscopy (ICP-AES, IRIS Intrepid II, Thermo Electron corporation, USA) as described [30].

### Isolation of bacteria

A sample of 5 grams of soil was suspended in 45 ml sterile water with glass beads. After shaking for 30 minutes and letting settle for 5 minutes, 200 µl of the liquid phase was inoculated on beef extract-peptone agar medium (beef extract 3.0 g l^−1^, peptone 10.0 g l^−1^, NaCl 5.0 g l^−1^, agar 18.0 g l^−1^, pH 7.0) in Petri dish (90 mm diameter×10 mm depth). Isolates were selected based on differences in colony morphology and re-streaked several times on beef extract-peptone agar at 28°C until 136 pure cultures were obtained.

### Genetic identification of isolated bacteria

Total DNA was extracted by the phenol-chloroform method as described [31]. To group the 136 isolates, BOX-PCR with the primer BOXA1R (5′-CTACGGCAAGGCGACGCTGACG-3′) was carried out as described [32], [33]. A dendrogram based on the BOXA1R-PCR fingerprints was drawn using Numerical Taxonomy and Multivariate Analysis System NTSYSpc 2.2 (Exeter Software, USA). An isolate from each of the 91 BOXA1R-PCR groups was chosen for 16S rRNA gene sequencing. Almost full length 16S rRNA gene was amplified by polymerase chain reaction (PCR) with the universal primers of 27F (5′-AGAGTTTGATCCTGGCTCAG-3′) and 1492R (5′-GGTTACCTTGTTACGACTT-3′) [34], [35] and sequenced at the Beijing Genomics Institute (Shenzhen, China). The sequences were submitted GenBank to assign accession numbers. The closest matching sequences were searched from GenBank with BLAST [36]. A neighbor joining the 16S rRNA phylogenetic tree was constructed using the neighbor joining method in MEGA 6.0 [37].

### Heavy metal tolerance tests

The resistance of the 136 isolates to lead, cadmium, zinc, copper, cobalt and nickel was assayed by spot-inoculating 10 µl of 10^8^ cells ml^−1^ bacterial suspension on beef extract-peptone agar medium with the respective metal salts. Pb(NO_3_)_2_, CdCl_2_, ZnCl_2_, CuSO_4_, CoCl_2_ and NiCl_2_ were added to the medium to obtain 200, 400, 600, 800 and 1000 mg kg^−1^ heavy metal concentrations. After incubation at 28°C for 5 days, the minimum inhibitory concentration (MIC) was defined as the lowest concentration of metal salt inhibiting bacterial growth. On the positive control plates without heavy metal the colonies were approximately four mm in diameter.

### Indoleacetic acid and siderophore production assays

The plant growth-promoting activity of the isolates was evaluated by assaying the production of indole-3-acetic acid (IAA) and siderophores as described. In the qualitative IAA assay [38], isolates were grown in a beef extract-peptone liquid medium with 0.5 g l^−1^ tryptophan at 28°C and 140 rpm for 36 hours, 50 µl of the culture suspension was absorbed into a white porcelain board and, after adding 100 µl of the color reagent (4.5 g l^−1^ FeCl_3_, 57.6% H_2_SO_4_), the board was incubated at 25°C for 30 min. A pink color indicated positive IAA production. A non-inoculated beef extract-peptone liquid medium with tryptophan served as a negative control. To quantify IAA production [38], [39], 4 ml of the color reagent was added to 2 ml of the culture supernatant obtained by centrifugation (8000 rpm for 5 min). Optical density at 550 nm was measured by spectrophotometry (WFJ2100, UNICO, China) after coloration for 30 min. IAA concentration in the supernatant was interpolated using an IAA standard curve. The distribution of IAA producers and heavy metal tolerant strains among the different taxa were compared with a Chi-square test.

In the qualitative siderophore assay, isolates were grown on chrome azurol sulphonate (CAS) agar to select siderophore producing strains [40]. To quantify siderophore production, siderophore producing strains were grown in Fiss minimal medium (5.03 g l^−1 ^L-asparagine, 5.03 g l^−1^ KH_2_PO_4_, 5.0 g l^−1^ glucose, 0.5 mg l^−1^ ZnCl_2_, 40 mg l^−1 ^MgSO_4_ and 0.5 µM FeSO_4_) for two days. After centrifugation at 1000 *g* for 15 min, supernatant was mixed with CAS solution (1 vol: 1 vol) and incubated for 60 min. Optical density at 400 nm was measured by spectrophotometry (WFJ2100, UNICO, China) [41]. Siderophore concentration in the supernatant was interpolated using a deferoxamine mesylate salt (SIGMA, USA) standard curve. The IAA and siderophore production and heavy metal tolerance assays were done in triplicate.

## Results

### Basic physicochemical properties of soil samples

To assess the quality of the V-Ti magnetite mine tailing soil, we first measured the soil physicochemical characteristics and heavy metal content in the soil (Table 1). The soil pH was low (5.28±0.91), as was the content of organic matter (16.98±4.45**‰**). Of the 103.50±36.84 mg kg^−1^ total N, approximately 13% was plant-available N. As expected, the iron, titanium and vanadium concentrations were high, up to 76.15, 28.19 and 5.58 g kg^−1^, respectively. The manganese concentration was 1.42 g kg^−1^. The concentration of chromium, zinc, copper and nickel were 98.51, 87.72, 56.75 and 48.11 mg kg^−1^. In addition, lead (3.87 mg kg^−1^), arsenic (0.94 mg kg^−1^) and Cadmium (0.52±0.25 mg kg^−1^) were detected in the mine tailing soil.

**Table 1:** The basic physicochemical properties and heavy metal concentrations in V-Ti magnetite mine tailing soil.

Properties	Average value	Minimum value	Maximum value
pH	5.28±0.91	4.48	6.34
Organic matter (‰)	16.98±4.45	12.72	22.29
Total N (mg kg^−1^)	103.50±36.84	72.85	153.73
Available N (mg kg^−1^)	13.64±8.03	8.46	25.60
Available K (mg kg^−1^)	11.71±3.86	7.21	15.98
Available P (mg kg^−1^)	11.08±1.82	8.87	13.15
As (mg kg^−1^)	0.94±0.58	0.47	1.77
Fe (mg kg^−1^)	76145.84±3715.20	70782.50	78647.50
Ti (mg kg^−1^)	28185.84±3264.46	23976.67	31596.67
V (mg kg^−1^)	5584.38±2457.28	2974.00	7595.00
Cr (mg kg^−1^)	98.51±9.20	90.61	111.72
Mn (mg kg^−1^)	1417.17±141.02	1268.33	1543.75
Zn (mg kg^−1^)	87.72±20.27	69.21	106.59
Cu (mg kg^−1^)	56.75±30.65	30.05	95.58
Ni (mg kg^−1^)	48.11±12.23	38.40	65.34
Pb (mg kg^−1^)	6.90±1.37	5.62	8.84
Cd (mg kg^−1^)	0.52±0.25	0.19	0.78

### Isolation of bacteria and genetic identification

**Figure 1:**
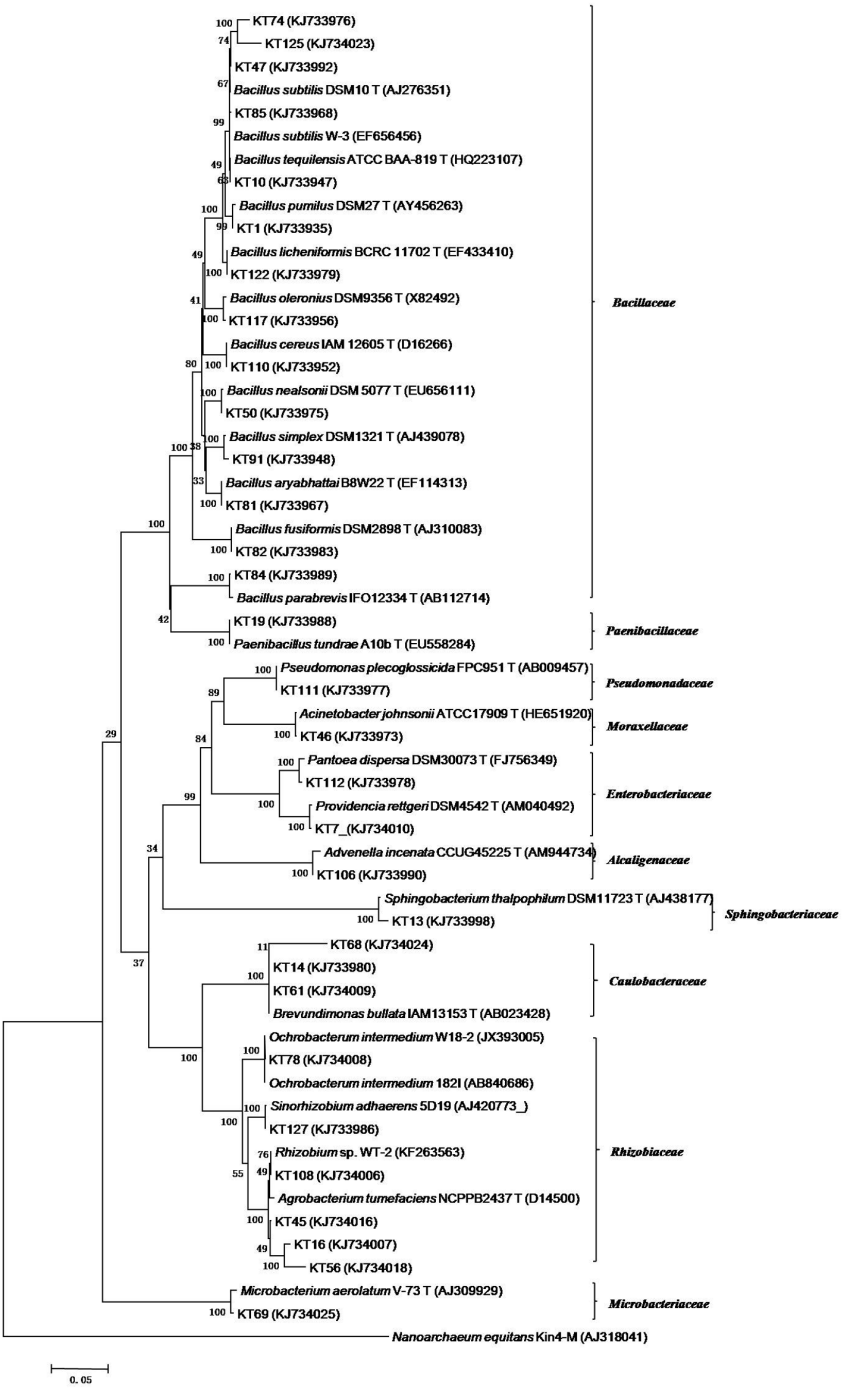
Neighbor-joining tree based on 16S rRNA sequences of isolated representative bacteria strains from V-Ti magnetite mine tailing soil by MEGA 6. The scale bar corresponds to 0.05 substitutions per nucleotide position. The numbers at nodes indicated the levels of bootstrap support (%) based on 1,000 resampled data sets; only values above 50% are given. Superscript “T” means type stains; Number in parentheses represents the sequence number in GenBank. *Nanoarchaeum equitans* Kin4-M was used as an outgroup.

Based on differences in colony morphology, 136 bacterial strains were isolated from the V-Ti magnetite mine tailing soil. According to the BOX A1R-PCR fingerprint analysis, the similarities between the 136 isolates ranged from 0.54 to 1.00. Altogether there were 91 distinct fingerprint patterns. The 136 isolates were divided into two major groups, group I (81 isolates) and group II (55 isolates), at 54% similarity level (Figure S1). One strain was chosen from each of the 91 distinct fingerprint pattern groups for 16S rRNA sequencing (Figure S1). The sequences were assigned GenBank accession numbers KJ733935–KJ734025 (Table S1). The16S rRNA gene sequences of the 91 representative strains indicated that the group I and group II in the BOX A1R-PCR dendrogram represented Gram-positive and Gram-negative bacteria, respectively (Table S1, Figure S1). Seventy-nine isolates belonged to the genus *Bacillus* and represented eleven species (Table S1). Altogether 32 isolates were considered as representing *B. subtilis* and 14 as *B. pumilus* (Figure 1, Table S1). In addition to the *Bacillus* spp. isolates, isolates KT19 and KT84 were identified as Gram-positive strains displaying 99% similarity to the type strains of *Paenibacillus tundrae* and *Microbacterium aerolatum*, respectively (Figure 1, Table S1).

The 55 Gram-negative isolates were assigned to seven families and ten genera (Figure 1, Table S1). Altogether 43 of them belonged to the *Rhizobiaceae* and were assigned as *Rhizobium* sp. (29 isolates), *Ochrobactrum intermedium* (13 isolates) and *Sinorhizobium adhaerens* (1 isolate).

### Heavy metal tolerance of isolated bacteria

**Figure 2:**
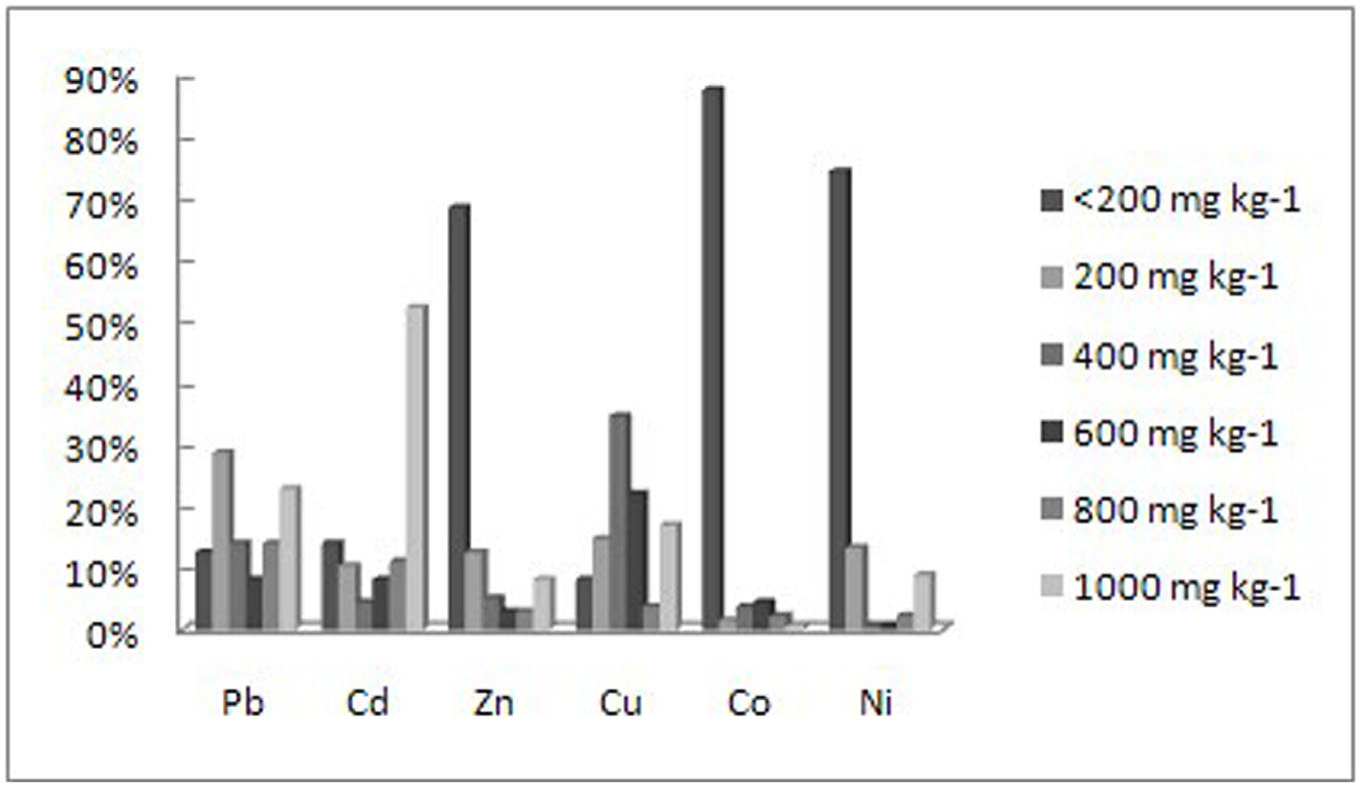
The minimum inhibitory concentrations (MICs) of bacterial isolates against six heavy metals. Pb, lead; Cd, cadmium; Zn, zinc; Cu, copper; Co, cobalt; Ni, nickel.

The heavy metal tolerance of the 136 bacterial isolates from the V-Ti magnetite mine tailing soil was determined as the minimum inhibitory concentration (MIC) (Figure 2). Most isolates showed MIC lower than 200 mg kg^−1^ for Zn (68.4%), Co (87.5%) and Ni (74.3%). Altogether 93 isolates tolerated the highest concentration (1,000 mg kg^−1^) of at least one tested heavy metal; 71 strains tolerated 1,000 mg kg^−1^ cadmium whereas only one strain, *Bacillus* sp. KT-76, tolerated 1,000 mg kg^−1^ cobalt. Only three strains, the *Rhizobium* sp. KT27 and KT62, and the *Bacillus* sp. KT43 displayed MIC less than 200 mg kg^−1^ for all the six heavy metals tested. Five strains, the *B. licheniformis* KT-87 and KT-88 and the *Bacillus* sp. KT-72, KT-74 and KT-76 were tolerant against all the tested heavy metals. *B. licheniformis* KT87 showed 1000 mg kg^−1^ MIC for Cd, Zn, Cu and Ni, 800 mg kg^−1^ MIC for Pb and 600 mg kg^−1^ MIC for Co. The MIC of *Bacillus* sp. KT72 for the six heavy metals was 1000 mg kg^−1^ (Pb, Cd, Ni) and 600 mg kg^−1^ (Zn, Cu, Co). When comparing the percentage of strains tolerant to four or more heavy metals, it was noted that, among the *Rhizobium* sp., multiple tolerant strains (34.9%) were less abundant than among the *Bacillus* spp. (54.4%) (p<0.05).

### Indoleacetic acid and siderophore production

Most of the isolates, altogether 91 strains, produced IAA (Table S1) ranging from 2.2 to 83.05 µg ml^−1^. Eleven strains produced more than 60 µg ml^−1^ IAA. Six of these were *Ochrobactrum* sp., three were *Bacillus* spp., one was *Providencia* sp. and one *Rhizobium* sp. *Ochrobactrum* sp. KT80 produced highest concentration (83.05 µg mL**^−^** ^1^) of IAA among the 136 isolates, followed by *Providencia* sp. KT7 (79.02 µg mL**^−^** ^1^) and *Rhizobium* sp. KT24 (75.82 µg ml^−1^).

Only 29 of the 136 isolates produced siderophore (Table S1) ranging from 5.50 to 167.67 µg ml^−1^. All the six *Rhizobium* sp. isolates that produced siderophore were IAA producers, whereas only half of the eighteen *Bacillus* spp. and two *Ochrobactrum* sp. siderophore producers produced IAA. *Bacillus* sp. isolates KT93 and KT113 that did not produce IAA produced highest siderophore concentrations of 167.67 µg ml^−1^ and 105.33 µg ml^−1^, respectively.

## Discussion

### Basic physicochemical properties of the V-Ti magnetite mine tailing soil

The basic physicochemical properties are important factors for evaluating soil quality. The V-Ti magnetite tailing, a weakly acid soil, showed pH similar to that in an old Spanish Pb-Zn mine soil [42]. Soil pH is the best predictor of microbial diversity and community composition [43], [44]. The bacteria in the V-Ti magnetite mine tailing soil may prefer acid environment. The organic matter content of V-Ti magnetite mine tailing soil was lower than in agricultural and urban ecosystem soils [29], [45], but similar with other mine tailing area [42]. The V-Ti magnetite mine tailing also showed lower content of available N, P and K than agricultural and urban ecosystem soils [29], [45], The low contents of available N, P, K and organic matter suggested that V-Ti magnetite mine tailing soil was unfertile. The iron, titanium and vanadium concentrations were up to three, ten and 70 times higher than in US soils in average [46], respectively. The concentrations of Cr, Zn, Cu, Ni and Mn were approximately 1.5 to 2.5 times higher than in US soils in average [46]. The concentrations of Fe, Cu and Zn were above values considered very high by Abreu *et al* [47] and the chromium concentration was almost twice as high as needed to inhibit alfalfa germination [48], plausibly explaining the scarce vegetation at the Zhujiabaobao V-Ti magnetite tailing dam. Therefore, phytoremediation of the barren V-Ti magnetite mine tailing soil should include increasing the content of available N, P, K and organic matter and lowing metal concentration.

### Genetic identification of bacterial isolates

To assess if the heavy metal-resistance and plant growth promoter-producing bacteria in the Zhujiabaobao V-Ti magnetite tailing dam soil would support phytoremediation, we isolated 136 bacterial strains, grouped them by BOX A1R-PCR and identified representative strains by 16S rRNA gene sequencing. The bacteria in the V-Ti magnetite mine tailing soil represented both Gram-negative and Gram-positive species. *Bacillus* spp. were the most abundant species, followed by *Rhizobium* spp. and *Ochrobactrum* spp. The spore and cyst forming capability of *Bacillus* spp. may explain why *Bacillus* spp. were abundant in the unfavorable environment of the V-Ti magnetite mine tailing soil. Autochthonous *Bacillus* from mine tailing in South Korea showed the ability to biomineralize heavy metals, such as Pb and Cr [49], [50]. *Ochrobactrum* spp. and *Pseudomonas* spp. have been used for the bioremediation of environmental pollutants [51]–[53]. These observations suggested that the indigenous bacteria might be useful for phytoremediation of the Zhujiabaobao mine tailing soil. The presence of multiple autochthonous *Rhizobium* spp. implied that, with compatible leguminous plants, the rhizobium-legume symbiosis could be used to gradually increase nitrogen content and overall fertility in the barren soil. The symbiosis of rhizobia and leguminous plants has been effectively used to remediate contaminated soil [54], [55].

### Heavy metal tolerance of bacterial isolates

To estimate the usefulness of the isolated bacteria in bioremediation, we assessed their heavy metal tolerance. Obviously, the bacteria in V-Ti magnetite mine soil have to tolerate the harsh environment polluted by heavy metals. The tolerance mechanisms include exclusion, extrusion, accommodation, bio-transformation and methylation or demethylation [13]. Bacteria can enhance metal solubility by producing acid and detoxify metals by removal, sequestering or immobilizing [56]. Since heavy metal tolerance is one of the most important factors for using an indigenous microorganism in bioremediation, recently the functional diversity of bacterial communities in contaminated soil has attracted more attention [7].

The bacterial isolates from V-Ti magnetite mine tailing soil showed diverse tolerance to different heavy metals. Most of the isolates did not tolerate the lowest tested concentration (200 mg kg^−1^) of Ni, Co and Zn. The concentrations of Ni and Zn were low in the mine tailing soil. A few of the V-Ti magnetite mine tailing isolates, e.g. *Bacillus* sp. KT87 and *Bacillus* sp. KT72, showed tolerance to higher metal concentrations than isolates from a copper mine tailing, from a mercuric salt-contaminated soil and from chickpea rhizosphere soil [57]–[59]. Interestingly, even though the concentration of Cd in the mine soil was not high, more than half the isolates tolerated a high concentration of Cd (1,000 mg kg^−1^). Likewise, even though the concentration of Pb in the mine tailing soil was low, some isolates tolerated a high concentration of Pb, i.e. the tolerance to heavy metals and the heavy metal content of soil did not directly correlate. Many mine tailing sites are polluted by multiple metals. For bacteria, the ability to survive, including variation in strains and characteristics, is related to environmental conditions and length of exposure to those conditions [60]. Therefore, the isolates from the V-Ti magnetite mine tailing soil showing multi-metal resistance were affected by the unfavorable environment. The culturable bacteria included isolates with multiple heavy metal tolerance, especially among the *Bacillus* spp., suggesting that the indigenous bacteria are capable of assisting the bioremediation of the V-Ti magnetite mine tailing soil polluted by heavy metals.

### Plant growth-promoting activity of bacteria

Plant-associated bacteria play a key role in host adaptation to changing environment by altering plant cell metabolism or promoting plant growth. Plant growth promoting rhizobacteria (PGPR) producing IAA and siderophore have been widely used to accelerate phytoremediation of metalliferous soil [13], [21]. Production of indoleacetic acid (IAA), a phytohormone, is a key characteristic of PGPR [61]. The addition of IAA to soil can enhance the uptake of metals in plant roots [13], [15], [62]. Even though PGPR are widely studied, few studies have systematically analyzed PGPR in contaminated soil. About 23% and 50% of Zn- and Cd-accumulating isolates from a former zinc and lead mining and processing site in Austria produced IAA and siderophore, respectively [63]. In V-Ti magnetite mine tailing soil the percentages of culturable rhizosphere IAA and siderophore producers were entirely different at 67% and 21%, respectively. The plant growth promoting activity of the isolates from the V-Ti magnetite mine tailing was stronger than that reported for *Pseudomonas putida* GR12-2 (IAA: 2.01 µg ml^−1^) from the rhizosphere of an arctic plant [64], but lower than that of *Alcaligenes faecalis* BCCM IC 2374 (Siderophore: 347 µg ml^−1^) [65], suggesting that the plant growth promoting activity from different environments is totally different. The abundance of isolates producing more than 20 µg ml^−1^ IAA suggested that the plant growth promoting ability of the isolates might assist in phytoremediating the soil.

The bacterial and fungal siderophores facilitate iron uptake in soil [10]. Iron chelated by siderophores is unavailable to plant pathogens resulting in an increase in plant health [13]. Metal–resistant siderophore-producing bacteria play important role in the successful survival and growth of plants in contaminated soil by alleviating metal toxicity and supplying nutrients for plant, and bacterial siderophore can bind metals other than iron [66], which may be the reason why microorganism can survive in the mine tailing soil contaminated by multi-metals. Bacterial siderophore should be beneficial to regulate availability of the abundant iron in the V-Ti magnetite mine tailing soil containing high concentration of iron. As bioaugmentation-assisted phytoextraction technology, the indigenous siderophore-producing bacteria can increase the phytoextraction rate that usually limits the use of phytoremediation methods [67]. Aside from their involvement in iron acquisition, siderophores have physiological roles of protecting some bacteria against the toxic effect of pyochelin by reducing reactive oxygen species [68], so the presence of siderophore-producing bacteria in the mine tailing can directly or indirectly promote bioremediation for the contaminated soil. Many isolates showed both IAA and siderophore production, implying that the characteristics of the indigenous bacteria are helpful in bioremediating the desert mine tailing area. Phytoremediation of metals was facilitated by PGPR by promoting plant growth and increasing the amount of metal taken up by plant [21], [69].

The heavy metal-resistance and plant growth-promoting activity are key characteristics for bacteria that are to be applied in metal phytoremediation. Therefore, analyzing these characteristics in an indigenous bacterial in contaminated sites is essential to provide significant information for developing effective bioremediation measures. Moreover, because both the structural and functional bacterial diversity are important indicators of soil health, evaluation for diversity of heavy metal-resistant bacteria and PGPR should be considered as the primary work for bioremediating soil contaminated by heavy metals. We showed that V-Ti magnetite mine tailing soil in Zhujiabaobao contained abundant bacteria that tolerate multiple heavy metals and have plant growth-promoting abilities. The results suggested that the indigenous bacteria in the soil have characteristics beneficial for remediating the contaminated mine tailing soil. To further study the phytoremediation approach, the plant growth promoting activity will be studied both in greenhouses and *in situ* in Zhujiabaobao.

## Supporting Information

Figure S1 **BOX-A1R dendrogram for 136 isolates from V-Ti magnetite mine tailing soil in Panzhihua, Sichuan, China.**
(TIF)

Table S1 **IAA, Siderophore production and preliminary BOX-AIR PCR identification of V-Ti magnetite mine tailing soil isolates, and similarity analysis of 16S rRNA sequence for 91 selected strains.**
(PDF)
