# Supplementary figures and images for: Culturable Heavy Metal-Resistant and Plant Growth Promoting Bacteria in V-Ti Magnetite Mine Tailing Soil from Panzhihua, China
Source: PLoS One. 2014 Sep 4;9(9):e106618. doi: 10.1371/journal.pone.0106618 (PMC4154735; doi:10.1371/journal.pone.0106618)

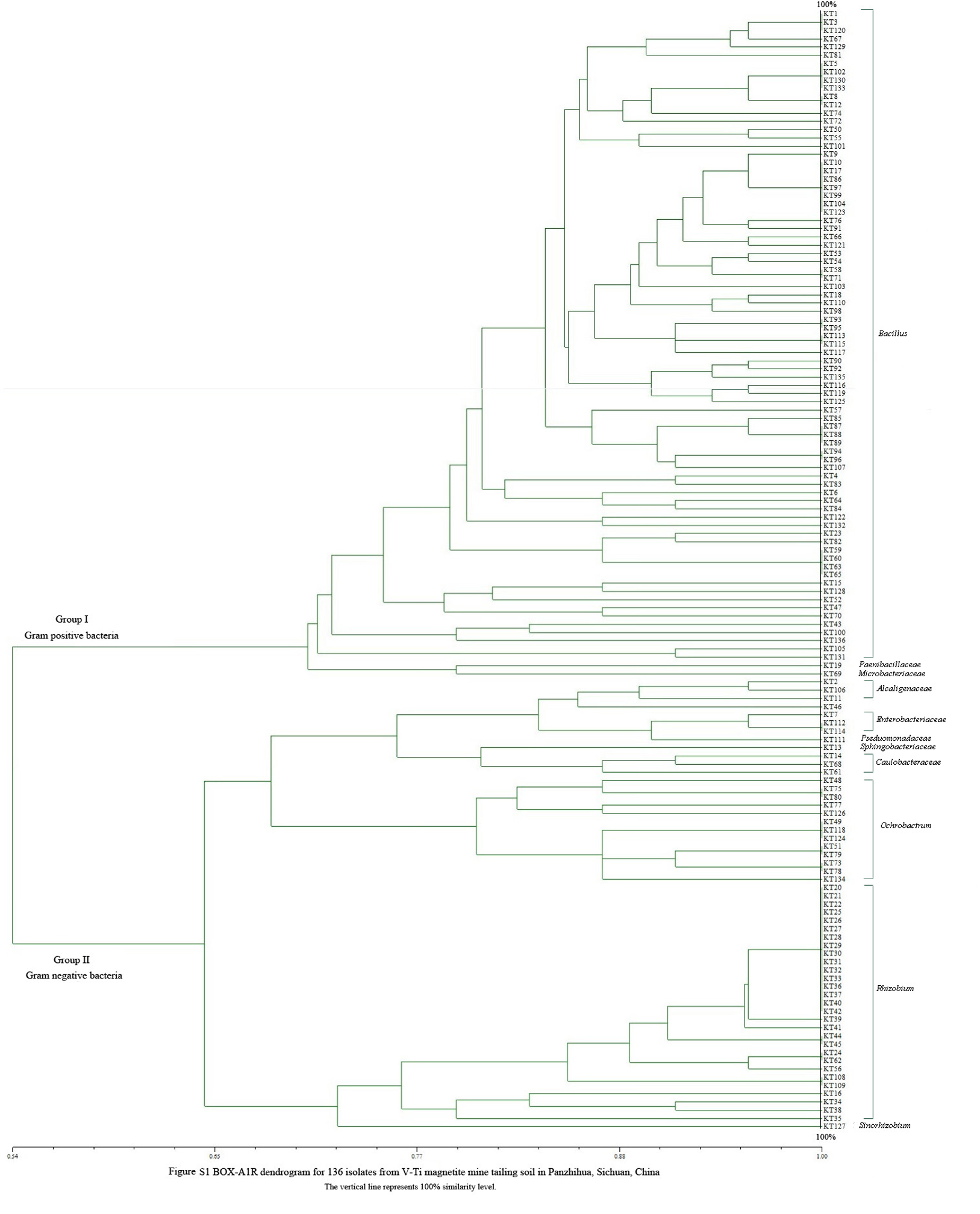

Supplement: Figure S1 — BOX-A1R dendrogram for 136 isolates from V-Ti magnetite mine tailing soil in Panzhihua, Sichuan, China. (TIF) [file pone.0106618.s001.tif]
