# Supplementary material for: Culturable Heavy Metal-Resistant and Plant Growth Promoting Bacteria in V-Ti Magnetite Mine Tailing Soil from Panzhihua, China
Source: PLoS One. 2014 Sep 4;9(9):e106618. doi: 10.1371/journal.pone.0106618 (PMC4154735; doi:10.1371/journal.pone.0106618)
Supplement: Table S1 — IAA, Siderophore production and preliminary BOX-AIR PCR identification of V-Ti magnetite mine tailing soil isolates, and similarity analysis of 16S rRNA sequence for 91 selected strains. (PDF) [file pone.0106618.s002.pdf]

Table S1. IAA, Siderophore production and preliminary BOX-AIR PCR identification of V-Ti magnetite mine tailing soil isolates, and similarity analysis of 16S rRNA sequence for 91 selected strains.

Family	Strain name	IAA ($\mu g\ ml^{-1}$)	Siderophore ($\mu g\ ml^{-1}$)	BOX-A1R Group	16S rRNA Length (bp)	16S rRNA GenBank NO.	16S rRNA blast analysis		
							Similar Genus	GenBank NO.	Similarity (%)
Bacillaceae	KT74	10.04±4.42	17.75±1.22	Group I	1429	KJ733976	*Bacillus subtilis*	JQ973708.1	99%
	KT125	15.71±4.56	0	Group I	1432	KJ734023	*Bacillus subtilis*	EF656456.1	97%
	KT47	0	0	Group I	1399	KJ733992	*Bacillus subtilis*	KF649246.1	99%
	KT6	0	0	Group I	1423	KJ733987	*Bacillus subtilis*	KF381492.1	99%
	KT116	55.82±3.87	82.08±12.62	Group I	1431	KJ733964	*Bacillus subtilis*	KC456632.1	99%
	KT66	8.65±2.37	14.92±1.74	Group I	1429	KJ733962	*Bacillus subtilis*	KF381492.1	100%
	KT135	18.80±0.82	0	Group I	1417	KJ733961	*Bacillus subtilis*	KJ081196.1	100%
	KT92	39.14±4.03	58.92±5.06	Group I	1456	KJ733960	*Bacillus subtilis*	JQ695930.1	99%
	KT93	0	167.67±32.76	Group I	1421	KJ733954	*Bacillus subtilis*	CP006881.1	100%
	KT95	0	0	Group I	-	-	-	-	-
	KT101	0	0	Group I	1430	KJ733953	*Bacillus subtilis*	KF818630.1	100%
	KT98	0	0	Group I	1463	KJ733951	*Bacillus subtilis*	FJ263021.1	99%
	KT53	0	0	Group I	1428	KJ733949	*Bacillus subtilis*	JN366715.1	99%
	KT9	2.20±0.54	0	Group I	1428	KJ733946	*Bacillus subtilis*	JF322922.1	99%
	KT12	0	61.25±5.73	Group I	1408	KJ733945	*Bacillus subtilis*	KC441757.1	99%
	KT8	9.15±0.40	0	Group I	-	-	-	-	-
	KT85	38.50±7.54	0	Group I	1418	KJ733968	*Bacillus subtilis*	CP006881.1	99%
	KT94	0	0	Group I	1421	KJ733972	*Bacillus cereus*	CP006881.1	100%
	KT96	0	0	Group I	-	-	-	-	-
	KT63	14.88±2.57	0	Group I	1416	KJ733981	*Bacillus subtilis*	KC441769.1	99%
	KT59	15.94±2.78	8.75±0.82	Group I	-	-	-	-	-
	KT60	8.00±2.53	0	Group I	-	-	-	-	-
	KT65	0	15.00±2.67	Group I	-	-	-	-	-
	KT23	34.28±4.43	0	Group I	1407	KJ733982	*Bacillus subtilis*	HQ418833.1	99%
	KT83	2.16±0.12	0	Group I	1430	KJ733985	*Bacillus subtilis*	HQ166109.1	100%
	KT64	3.26±0.36	0	Group I	1428	KJ733995	*Bacillus subtilis*	KC441757.1	100%
	KT100	0	0	Group I	1431	KJ734000	*Bacillus subtilis*	KF001839.1	100%
	KT105	0	0	Group I	1460	KJ734002	*Bacillus subtilis*	GU272021.1	99%
	KT71	0	0	Group I	1421	KJ734012	*Bacillus tequilensis*	KC172031.1	100%
	KT58	7.17±1.45	0	Group I	-	-	-	-	-

KT10	29.13±3.11	0	Group I	1454	KJ733947	*Bacillus tequilensis*	JF411314.1	99%
KT17	36.05±2.73	0	Group I	-	-	-	-	-
KT86	0	0	Group I	-	-	-	-	-
KT97	0	10.33±3.32	Group I	-	-	-	-	-
KT99	0	0	Group I	-	-	-	-	-
KT104	13.84±2.14	0	Group I	-	-	-	-	-
KT123	0	0	Group I	-	-	-	-	-
KT113	0	105.33±6.91	Group I	1419	KJ733955	*Bacillus subtilis*	CP006881.1	100%
KT115	0	0	Group I	-	-	-	-	-
KT103	10.41±1.70	19.17±1.83	Group I	1429	KJ733958	*Bacillus tequilensis*	JF411247.1	100%
KT107	0	20.08±1.12	Group I	1430	KJ733970	*Bacillus tequilensis*	JF411297.1	100%
KT52	0	9.08±2.06	Group I	1428	KJ733993	*Bacillus subtilis*	GQ262728.1	100%
KT132	0	0	Group I	1423	KJ733997	*Bacillus tequilensis*	JF411299.1	100%
KT122	0	0	Group I	1428	KJ733979	*Bacillus licheniformis*	KF535146.1	100%
KT70	0	0	Group I	1431	KJ734004	*Bacillus licheniformis*	JX027378.1	99%
KT89	0	0	Group I	1459	KJ733969	*Bacillus licheniformis*	GQ470399.1	99%
KT87	34.75±4.30	0	Group I	-	-	-	-	-
KT88	29.69±2.12	0	Group I	-	-	-	-	-
KT131	0	0	Group I	1409	KJ734003	*Bacillus licheniformis*	KC969075.1	99%
KT1	11.63±2.26	13.67±1.03	Group I	1426	KJ733935	*Bacillus pumilus*	KF933667.1	99%
KT3	0	22.00±4.10	Group I	-	-	-	-	-
KT120	0	10.75±1.47	Group I	-	-	-	-	-
KT76	10.68±0.84	0	Group I	1421	KJ733938	*Bacillus pumilus*	KF956587.1	100%
KT129	0	0	Group I	1429	KJ733940	*Bacillus pumilus*	GU593626.1	99%
KT133	0	0	Group I	1467	KJ733944	*Bacillus pumilus*	KC692160.1	99%
KT5	12.38±2.29	0	Group I	-	-	-	-	-
KT102	0	0	Group I	-	-	-	-	-
KT130	13.53±1.54	0	Group I	-	-	-	-	-
KT54	0	0	Group I	1455	KJ733950	*Bacillus pumilus*	KC953600.1	99%
KT90	61.83±4.92	6.42±0.85	Group I	1419	KJ733959	*Bacillus pumilus*	KC915229.1	100%
KT121	65.99±5.57	0	Group I	1430	KJ733963	*Bacillus pumilus*	GU593626.1	99%
KT4	0	0	Group I	1426	KJ733984	*Bacillus pumilus*	EF091147.1	98%
KT119	0	0	Group I	1427	KJ733965	*Bacillus pumilus*	KF158229.1	99%
KT72	32.47±3.31	9.33±1.50	Group I	1429	KJ733996	*Bacillus pumilus*	KF158227.1	100%
KT117	5.85±2.29	0	Group I	1440	KJ733956	*Bacillus oleronius*	JN366717.1	99%

	KT110	0	0	Group I	1420	KJ733952	*Bacillus cereus*	KF601957.1	100%
	KT136	6.53±0.57	0	Group I	1429	KJ734001	*Bacillus cereus*	KF933614.1	100%
	KT18	16.44±3.26	0	Group I	1428	KJ734022	*Bacillus cereus*	KF500919.1	100%
	KT50	74.92±3.51	0	Group I	1426	KJ733975	*Bacillus nealsonii*	KF535131.1	99%
	KT43	38.02±7.08	0	Group I	1435	KJ733999	*Bacillus nealsonii*	KC329823.1	99%
	KT57	9.75±1.08	0	Group I	1432	KJ733966	*Bacillus nealsonii*	KF535131.1	99%
	KT67	28.44±1.19	0	Group I	1428	KJ733936	*Bacillus nealsonii*	KF535131.1	100%
	KT81	19.79±3.31	0	Group I	1397	KJ733967	*Bacillus aryabhattai*	HG424432.1	100%
	KT55	48.10±5.32	0	Group I	1457	KJ733974	*Bacillus aryabhattai*	JF895478.1	99%
	KT91	0	0	Group I	1405	KJ733948	*Bacillus simplex*	KF818647.1	99%
	KT128	16.08±3.18	0	Group I	1437	KJ733994	*Bacillus simplex*	JF496504.1	99%
	KT15	10.39±1.20	0	Group I	1436	KJ734005	*Bacillus simplex*	JF496323.1	99%
	KT82	29.60±3.03	0	Group I	1419	KJ733983	*Bacillus fusiformis*	AB167231.1	99%
	KT84	7.55±1.62	0	Group I	1400	KJ733989	*Bacillus parabrevis*	JF460744.1	99%
Paenibacillaceae	KT19	10.95±2.40	0	Group I	1429	KJ733988	*Paenibacillus tundrae*	KF054885.1	99%
Microbacteriaceae	KT69	18.79±2.50	0	Group I	1394	KJ734025	*Microbacterium aerolatum*	JN934389.1	99%
Caulobacteraceae	KT68	8.83±2.00	0	Group II	1332	KJ734024	*Brevundimonas bullata*	EU977636.1	94%
	KT14	8.82±2.51	0	Group II	1328	KJ733980	*Brevundimonas bullata*	EU977700.1	99%
	KT61	0	0	Group II	1334	KJ734009	*Brevundimonas bullata*	EU977700.1	99%
Rhizobiaceae	KT78	69.03±3.85	0	Group II	1366	KJ734008	*Ochrobactrum intermedium*	AB840696.1	100%
	KT73	61.11±2.47	0	Group II	-	-	-	-	-
	KT48	21.00±1.68	0	Group II	1358	KJ734021	*Ochrobactrum intermedium*	JX393005.1	99%
	KT126	24.22±1.22	0	Group II	1359	KJ733991	*Ochrobactrum intermedium*	JX393005.1	100%
	KT77	71.23±5.07	14.58±3.98	Group II	1355	KJ733971	*Ochrobactrum intermedium*	JX393005.1	100%
	KT49	68.13±3.59	0	Group II	1391	KJ733957	*Ochrobactrum intermedium*	KF381492.1	100%
	KT118	0	13.42±3.78	Group II	-	-	-	-	-
	KT124	13.82±2.10	0	Group II	-	-	-	-	-
	KT134	0	0	Group II	1371	KJ733942	*Ochrobactrum intermedium*	JX393005.1	99%
	KT51	68.97±5.47	0	Group II	1302	KJ733941	*Ochrobactrum intermedium*	AB840686.1	99%
	KT79	26.86±4.14	0	Group II	-	-	-	-	-
	KT75	42.41±6.66	0	Group II	1356	KJ733937	*Ochrobactrum intermedium*	JX393005.1	100%
	KT80	83.05±5.61	0	Group II	-	-	-	-	-
	KT127	8.36±4.90	0	Group II	1362	KJ733986	*Sinorhizobium adhaerens*	AJ420773.1	99%
	KT108	8.68±3.32	0	Group II	1389	KJ734006	*Rhizoubium sp.*	HM151908.1	99%
	KT109	0	0	Group II	-	-	-	-	-

	KT35	41.15±0.86	0	Group II	1391	KJ734011	*Rhizobium sp.*	KF263563.1	99%
	KT21	28.16±2.48	28.58±2.58	Group II	1363	KJ734013	*Rhizobium sp.*	HM151908.1	99%
	KT20	36.11±1.26	0	Group II	-	-	-	-	-
	KT22	36.23±5.39	0	Group II	-	-	-	-	-
	KT25	44.68±3.96	0	Group II	-	-	-	-	-
	KT26	52.22±4.45	24.58±3.12	Group II	-	-	-	-	-
	KT27	29.95±0.45	34.75±4.64	Group II	-	-	-	-	-
	KT28	41.43±2.92	0	Group II	-	-	-	-	-
	KT29	29.81±3.22	46.92±4.48	Group II	-	-	-	-	-
	KT30	46.11±0.76	0	Group II	-	-	-	-	-
	KT31	29.69±3.72	0	Group II	-	-	-	-	-
	KT32	38.52±5.27	0	Group II	-	-	-	-	-
	KT33	36.11±5.45	0	Group II	-	-	-	-	-
	KT36	18.75±1.94	0	Group II	-	-	-	-	-
	KT37	43.30±3.38	0	Group II	-	-	-	-	-
	KT40	53.38±4.10	0	Group II	-	-	-	-	-
	KT42	41.89±7.81	0	Group II	-	-	-	-	-
	KT39	43.16±5.21	0	Group II	1399	KJ734014	*Rhizobium sp.*	KF263563.1	99%
	KT41	37.54±1.45	0	Group II	1365	KJ734015	*Rhizobium sp.*	KF008226.1	100%
	KT24	75.82±3.62	0	Group II	1394	KJ734017	*Rhizobium sp.*	HM756197.1	100%
	KT62	44.47±2.50	0	Group II	-	-	-	-	-
	KT34	47.71±3.52	37.75±0.89	Group II	1367	KJ734019	*Rhizobium sp.*	KF263563.1	99%
	KT38	37.05±3.93	0	Group II	1392	KJ734020	*Rhizobium sp.*	HM151908.1	100%
	KT45	38.15±3.47	0	Group II	1365	KJ734016	*Rhizobium sp.*	KF870446.1	9-9%
	KT44	46.82±3.27	0	Group II	-	-	-	-	
	KT16	23.99±3.10	33.83±1.20	Group II	1357	KJ734007	*Rhizobium sp.*	HG518323.1	98%
	KT56	54.47±4.29	0	Group II	1357	KJ734018	*Rhizobium sp.*	KF263563.1	97%
Alcaligenaceae	KT106	0	0	Group II	1407	KJ733990	*Advenella incenata*	KF844056.1	99%
	KT11	4.63±2.27	22.42±5.00	Group II	1397	KJ733939	*Advenella incenata*	KF844056.1	99%
	KT2	12.04±1.25	0	Group II	1408	KJ733943	*Advenella incenata*	KF844056.1	100%
Pseduomonadaceae	KT111	0	0	Group II	1414	KJ733977	*Pseudomonas plecoglossicida*	EU594553.1	99%
Moraxellaceae	KT46	19.03±2.96	5.50±0.62	Group II	1412	KJ733973	*Acinetobacter johnsonii*	KC895498.1	99%
Enterobacteriaceae	KT112	0	76.58±6.61	Group II	1414	KJ733978	*Pantoea dispersa*	GQ200831.1	99%
	KT114	5.36±0.96	0	Group II	-	-	-	-	-
	KT7	79.02±9.35	0	Group II	1360	KJ734010	*Providencia rettgeri*	KF923809.1	99%

Sphingobacteriaceae	KT13	0	0	Group II	1408	KJ733998	*Sphingobacterium thalpophilum*	HM771694.1	99%

The isolates with no accession number were identified by BOX-AIR PCR only.
